# Proposed Central Board

**Published:** 1892-07-30

**Authors:** 


### THE LONDON HOSPTALS.

### PROPOSED CENTRAL BOARD.

On the 22nd inst., at the invitation of Lord Sandhurst, a meeting of those interested in the management of hospitals and dispensaries was held at Spencer House, St. James's, to consider the conclusion arrived at by the Committee of the House of Lords on Metropolitan Hospitals—namely, that a central board should be created and receive a charter entitling it to receive endowments, legacies, bequests, and contributions for distribution to medical charities and to meet its own necessary expenses. The Lord Mayor presided, and amongst those present were Lord Sandhurst, Sir Rutherford Alcock, Sir Douglas Galton, Sir Sydney Waterlow, the Rev. Dr. Wace, Mr. Henry Lucas, Mr. Henry C. Burdett, Mr. G. F. Sheppard, Mr. W. Bousfield, Mr. J. A. Shaw Stewart, Mr. F. E. Villiers, Mr. Malcolm Morris, and Mr. R. Ackland.

The CHAIRMAN moved that Mr. P. Michelli (Seamen's Hospital) and Mr. C. L. Todd (St. George's Hospital) be appointed the Hon. Secretaries of the Conference.

Lord SANDHURST seconded, and the motion was carried.

The CHAIRMAN said that for nearly three sessions of Parliament a Committee of the House of Lords, presided over most ably by Lord Sandhurst, had made an inquiry into the working of the London hospitals. The conclusion they came to was that a central voluntary board should be formed for all the metropolitan hospitals. All were agreed on one point, viz., that the voluntary system in the cause of charity was the right one, and what they had to consider was not only how that voluntary system could be maintained, but how it could be still further assisted and made for all time sounder, if possible, than it was at the present time. The Hospital Sunday and Saturday Funds movement resulted every year in a very substantial sum of money, yet it did not entirely meet with all the requirements of the case. He could not venture to say at the moment as to whether the 1892 Sunday collections would equal those for the previous year, possibly not by some few thousands, but at all events these collections were always an uncertain source of income. It had struck him, and no doubt had struck many present, that if from the governing bodies of the hospitals of the metropolis a Voluntary Committee could be formed, each hospital sending or nominating as their representative a member or members of its governing body, it would go greatly to strengthen the position of the voluntary medical charities. They did not want State or municipal interference. (Hear, hear). They were all agreed on that point. If they could, therefore, form a Voluntary Committee, as he believed they would be disposed to do, to inquire into the general working of the hospitals of London—a Committee that would recognise fully the strength as well as the weakness of the working of the hospitals—so as to be able to at once put a finger upon any defect, and by suggestion, and possibly later on by a certain amount of control, to strengthen the position where it was weak, doing away with hospitals where they were not required, or where extended requirements were necessary adding to their number, he thought they would gather round the hospitals increased public confidence and be more assured from State interference than possibly would otherwise be the case. (Hear, hear.)

Lord SANDHURST thought it would be only right that hospital managers should give the suggestions contained in the Lords' Committee's report their most careful consideration. He had himself been at some little trouble to consider what would be the best way to give some practical result to the inquiry, of which for over two years he had been chairman. First he had thought it might be desirable to ask the Lord Mayor to convene a general meeting of the authorities to discuss the report, but on second thoughts he had found that would be attended with much inconvenience in that they would have a body of indefinite numbers, a general conversation, and probably no result. He had, therefore, decided first of all to call the smaller meeting they had that day, and, after due deliberation, to report to a general meeting of the London hospital managers. In the proposition he had to lay before them he did not ask the meeting to vote off-hand that it would be a wise thing to have a Central Board, but all he asked them to do was to elect a committee to thresh out the plan suggested by the Lords' Committee. After every possible consideration, they could then report the result of their consultation to that conference, who would express an opinion on the report, and, supposing they agreed to come into line, the Lord Mayor might be asked to convene a meeting of all the charities of London and see whether a definite plan could not be arrived at. He only asked them to say whether the plan laid down by the Lords' Committee was a good one, or whether it could be improved upon, and then to lay their report before the hospital boards for their consideration. As to the Committee, he had jotted down a few names to submit to them, including representatives of the Hospital Sunday and Saturday Funds, and at the same time he thought it would be an advantage if they could find some really good, strong outside man who would bring an impartial mind to bear on the deliberations of that committee. He had got in his mind one gentleman, a man of very great power—Mr. Diggle, the Chairman of the London School Board. He was a good organiser, a man who took a great interest in that question, and one who, he believed, if asked, would serve, and prove a very hard-working colleague. One of the reasons why he had called that meeting was that he was very anxious to see an attempt to organise the medical charities come from within themselves instead of being forced upon them by the public. All of them knew the great difficulty of procuring funds. Two or three hospitals during the past year had, owing to the receipt of large legacies, been placed in a fairly flourishing condition, but most of the medical charities had been hardly able to make both ends meet. Should such a state of things continue a great many of the hospitals would be obliged to close some of their beds. The municipal authorities might then say: "These institutions are very good as far as they go, but they have no money, and have to keep many beds closed; therefore we think we had better take them over and supply the necessary funds." What he particularly desired to do was to increase the public's confidence in the management of hospitals. That entire confidence was not placed in them by the general public was shown by the fact that the annual subscriptions in most cases were not sufficient even to pay the salaries of the staff. As a remedy for this the Lords' Committee had suggested the establishment of a Central Board. One of the criticisms on the idea was that such a board would do away with the individuality of hospitals, and that something like the *bureau centrale* of Paris would be the result. The Committee, however, in their report, said that they thought that very individuality was a good thing—a thing they wished to see preserved. Such criticism, therefore, fell to the ground. One great reason for the appointment of a Central Board was this. Oftentimes hospitals were built where they were not required, or located in incommodious buildings. It was true that it was not suggested that any statutory powers should be given to that board; but he considered the dictum of such a board would carry great weight. He had heard that there was a certain amount of opposition to that idea on the part of the medical side of the hospitals. To his mind, that side was not less interested in keeping up the voluntary system than the other. At the present time it was the wish of the Committee of every general hospital to provide, when asked by the medical staff, everything within reason that would tend to promote medical science. The medical profession would, he was afraid, find very much greater difficulty in getting their wants attended to by boards of guardians. When the time came for the hospitals to be taken over by the State or by the municipality (which time, he hoped, would be indefinitely postponed), he was sure that the first thing and the first people to suffer would be medical science and the medical profession. Such a Central Board would, he thought, tend to bring about co-operation between hospitals and hospitals, and hospitals and dispensaries, and possibly between hospitals and the poor law. In considering the resolution he had to propose, he begged his hearers to remember the difficulty of getting funds, and the number of beds which were being kept vacant owing to a want of funds. He moved "That a committee of this conference be appointed to take into consideration the conclusions arrived at by the Lords' Committee on Metropolitan Hospitals, and to report as to the composition and duties of the proposed voluntary Central Board, or such other scheme as may be arrived at after due deliberation." (Cheers.)

Sir Rutherford Alcock seconded the motion. Of all the suggestions made in the Lords' Report, that of a Central Board would be the one on which there would be the greatest diversity of opinion. Anything, however, like State control, anything like support from the State or the rates, would have the direct effect of destroying that which was really the pride of the metropolis, the enormous voluntary effort which is made annually to support the medical charities. To the putting of the charities under any absolute control which would interfere at all with the interests of the very large number of persons who have thrown themselves into the work and administration of hospitals, the objection would also be deep-rooted and difficult to overcome. Nevertheless it would be most desirable if some such scheme as a Central Board was practicable to establish such a board.

Sir Douglas Galton moved that the words in the resolution after "Metropolitan Hospitals" should be omitted, and that there should be substituted for them, "and to report to a future conference." He thought Lord Sandhurst's resolution took it too much for granted that a Central Board was to be formed. He confessed he viewed with very great apprehension the formation of such a board for the inspection of the hospitals. It would tend to be a board of control, and in that way it would, he was convinced, tend to diminish the interest at present felt by those gentlemen who took part in the management of their hospitals. Another objection would be that any answers made by a hospital to the criticisms of such a Board would necessarily be comparatively unknown to the public. Such a fact would materially diminish the funds of a hospital which was adversely criticised. Then again the receipt of bequests or money by such a body would rob giving of all sentiment, and so again would tend to diminish the amount of money obtained by the public.

Dr. F. W. Hunt seconded the amendment.

Sir Sydney Waterlow supported the amendment. He thought a Board without statutory powers was a form of machinery which it was very unwise to put into operation.

Lord Sandhurst adopted the amendment, which he said would enable the committee to take the whole of the Lords' recommendations into consideration. As to there being no statutory power, the Lords' Committee took its cue entirely from the Hospital Sunday Fund system.

Mr. Bousfield thought the Central Board would rely solely for its power on the justice and wisdom of the criticisms it made. He felt sure that the public would value the help and advice of such a body.

Mr. Burdett pointed out that, whatever certain gentlemen who had not given time and attention to the administration of hospitals might think, all those who had devoted themselves to the hospital cause felt as one man that any substitution of rate aid for the existing voluntary system, would be a great injustice and cruelty.

The motion was subsequently passed in the following altered form: "That a committee of this conference be appointed to take into consideration the conclusions arrived at by the Lords' Committee on Metropolitan Medical Charities, and to report to a future conference."

The following committee was appointed: Sir Sydney Waterlow (Hospital Sunday Fund), Mr. R. D. B. Acland (Hospital Saturday Fund), Sir Douglas Galton, Mr. Bousfield, Mr. J. Shaw Stewart, Mr. Lucas, Mr. Henry C. Burdett, Sir Andrew Clark, and Mr. Diggle, with power to add to their number.

Votes of thanks to the Lord Mayor for presiding, and to Lord Spencer for allowing the meeting to be held at his house, were passed, and the meeting separated.

